# Sea-Tangle, (Laminaria Digitata)—Its Use in the Treatment of Flexions of the Uterus

**Published:** 1864-12

**Authors:** M. S. Buttles

**Affiliations:** New York City


ART. II.—*Sea-Tangle, (Laminaria Digitata)—Its use in the treatment of Flexions of the Uterus, by* M. S. BUTTLES, M. D., *New York City.*

The *Laminaria Digitata* was first brought before the notice of the profession by Dr. Sloan of Ayr, in the *Glasgow Medical Journal* for October, 1862. In the *Medical Times and Gazette* of November 28, 1863, is an article by Dr. J. G. Wilson, in which he calls attention to its value in dilating the urethra and os uteri. Mr. Critchett also mentions it in the treatment of stricture of the lachrymal duct in an article, published in the *Lancet* of February 6, 1864.

It possesses many very important advantages; it can be worked up of any size and length; it is readily made smooth when dry, and is quite firm, yet elastic enough for all practicable purposes, so that it can be passed as readily as a silver probe. When exposed to moisture it expands to about four times its former size.

While making some experiments with this sea-weed I discovered that if a bougie made of it was bent while dry it would remain so until it was moistened, when it would gradually resume its former straightness; from this I coceived the idea of using it in the treatment of that frequent and hitherto obstinate displacement of the uterus known as flexion, whereupon I immediately gave it a trial, and the result thus far is of the satisfactory nature.

*Case*—Mrs. C——— had been troubled for twelve years with dysmenorrhœa, arising from flexion of the cervix uteri. So severe were her pains at each menstrual period that she was obliged to take her bed. The ordinary treatment at the hands of several skillful practitioners had given her no relief,

August 10, 1864, three days previous to her expected menstrual flow, I introduced a bougie made of the dried stem of the *Laminaria Digitata*, the size of a crow's quill, first warming it a little so as to make it flexible; this is best done by immersing in hot water, then bending it to the arc of a two-inch circle so as to enable me to pass it beyond the point of constriction, which was easily accomplished, and the bouge left in situ for twelve hours. When removed it was found to be perfectly straight, and three or four times its former size; there was not the slightest flexion of the cervix remaining.

The patient menstruated three days subsequent for the first time in her life without pain. The flexion partially returned, but the repetition of the treatment for the three succeeding months has entirely and I think permanently affected a cure. I have tried it in several similar cases with like good results, and hope the profession will take advantage of the above suggestions and give it a more thorough trial than I have been able to do. Several of our surgical instrument makers have sent to Europe for the Sea-Tangle, and will soon be able to supply those who may want it.

